# Supplementary material for: The discovery of novel noncoding RNAs in 50 bacterial genomes
Source: Nucleic Acids Res. 2024 Apr 22;52(9):5152–65. doi: 10.1093/nar/gkae248 (PMC11109978; doi:10.1093/nar/gkae248)
Supplement: gkae248_Supplemental_Files [file gkae248_supplemental_files.zip › Narunsky 50 Genomes RNA Motifs Files.zip!/Publication/56-D_turgidum/Figures/LRC-56-25-NC_011661_1_IGR_74_1747517_1747772.sto.pdf]

LRC-56-25-NC_011661_1_IGR_74_1747517_1747772.sto.r2r

5′-AACAACCCCCUCUUCUAYAGAA●YUGAAYC●●●A○○○○AAACUYA○○○Y●AACUU●●RR●●ARAA●AYCUCCYUCCYUUARGRAAAAC●AUCAURCAAUCGUUUYAAUAAUAUUAUAACUAAA●UU●●AA●UGUGUCAAUYAARRY○AAAAGUUUA●YUAUGAAAYUAARUU●●●Y●RRUUUGACAAURR●AA●●AGAAGRRAGUAUAAUUUAY○○○AA●A●●UUUUGGUUAUUUUGGAUAUUUAGGGGAGGUGRACU
